# Challenges and opportunities to provide immunization services: Analysis of data from a cross‐sectional study on a sample of pharmacists in a developing country

**DOI:** 10.1002/hsr2.1206

**Published:** 2023-04-13

**Authors:** Fouad Sakr, Mariam Dabbous, Mohamad Rahal, Pascale Salameh, Marwan Akel

**Affiliations:** ^1^ School of Pharmacy, Lebanese International University Beirut Lebanon; ^2^ École Doctorale Sciences de la Vie et de la Santé, Université Paris‐Est Créteil Créteil France; ^3^ UMR U955 INSERM, Institut Mondor de Recherche Biomédicale, Université Paris‐Est Créteil Créteil France; ^4^ INSPECT‐LB (Institut National de Santé Publique, d’Épidémiologie Clinique et de Toxicologie‐Liban) Beirut Lebanon; ^5^ School of Medicine, Lebanese American University Byblos Lebanon; ^6^ School of Pharmacy, Lebanese University Beirut Lebanon; ^7^ Department of Primary Care and Population Health University of Nicosia Medical School Nicosia Cyprus; ^8^ School of Education, Lebanese International University Beirut Lebanon

**Keywords:** barriers, experience, immunization, pharmacists, vaccines

## Abstract

**Background and Aims:**

Vaccine‐preventable illnesses continue to be a global health concern as immunization coverage remains below its targets. National plans emphasize on the essential role of multidisciplinary efforts and approaches to vaccination programs. Pharmacists are globally getting involved in immunization services as important members of the healthcare team. This study aimed to determine barriers, and assess challenges and possible opportunities to provide immunization in the Lebanese pharmacy practice.

**Methods:**

This was a cross‐sectional study that included pharmacists from all over Lebanon, as part of a national research to assess the role of pharmacists as immunizers. All registered pharmacists in Lebanon practicing in community, hospital, or other clinical settings were considered eligible for participation. A web‐based self‐administered validated questionnaire, which is initially developed by the American Pharmacists Association, was adapted with permission.

**Results:**

A total of 315 pharmacists responded to the survey. Only 23.1% declared completing an immunization training program. Over half of pharmacists (58.4%) administer vaccines to patients. A significant association between lack of support from physicians to pharmacists (adjusted odds ratio [ORa] = 2.099, 95% confidence interval [CI] = 1.290–3.414, *p* = 0.003) and vaccine administration was found, while cost associated with professional development and additional training (ORa = 0.533, 95% CI = 0.287–0.989, *p* = 0.046) was inversely associated to it. Logistic, financial, and legislative requirements as essential necessities were determined to successfully expand pharmacist‐led immunization services.

**Conclusions:**

The major barriers and challenges to vaccine administration by pharmacists comprised a lack of physicians' support and expenses associated with professional development and additional training. Pharmacists administer more vaccination despite lack of support from physicians; whereas they administer less vaccination due to cost associated with professional development and further training. The scope of pharmacy practice in Lebanon is not well recognized by other healthcare providers and stakeholders to include immunization services.

## 1 INTRODUCTION

Vaccination is one of the safest, most effective and economically proficient measures to prevent, eradicate and control life‐threatening infections.^1^ Vaccines are simply defined as biological substances that stimulate the development of immunoglobulins to protect against a disease.^2^ In fact, ordinary infections were a primary cause of global mortality before the introduction of vaccines.^3^, ^4^ The annual mortality prevention due to immunization is projected to be between 2 and 3 million deaths.^5^ There is a strong evidence that national immunization programs have a significant effect on the prevalence of infectious diseases.^6^ In the developed countries, immunization programs, and easier access to vaccination services have led to a very low rates of vaccine‐preventable illnesses.^7^ Nevertheless, vaccine‐preventable illnesses continue to be a global health concern as immunization coverage remains below its targets.^8^, ^9^, ^10^ To fully address this issue, national plans emphasize on the essential role of multidisciplinary efforts and approaches to vaccination programs. Healthcare professionals, principally primary care providers and pediatricians, are requested to collaborate in immunization programs to disseminate evidence‐based information about vaccines safety and efficacy, as well as the risks of vaccine‐preventable conditions.^11^

The role of pharmacists is constantly evolving as they are globally getting involved in immunization services in addition to being drug specialists, first‐line healthcare practitioners, and important members of the healthcare team.^12^, ^13^, ^14^ During the last 5 years, the International Pharmaceutical Federation (FIP) has led various surveys and produced several reports. The 2020 report revealed that the number of responses to its vaccination surveys has doubled since its last dissemination among its member organizations, indicating that the process of pharmacist involvement in vaccination is progressing.^15^ Moreover, a pharmacist recommendation to obtain immunization has been found to have a beneficial influence on a person's decision to become vaccinated.^16^ Pharmacists can increase vaccination uptake by playing the role of an educator, facilitator, and administrator and thus improving vaccine confidence and coverage in the community.^17^, ^18^

In the United States (US), pharmacist immunizers have verified the pursued benefits reinforcing the expansion of pharmacy practice to incorporate immunization.^19^, ^20^, ^21^ Although they encountered challenges and several barriers to administer vaccines relating to logistics and financial resources.^22^, ^23^, ^24^ On the other hand, important facilities were recognized including managerial and training support, and additional human resources to provide vaccination in the practice settings.^25^

Lebanon is a developing low‐ middle‐income country that is located in the Middle East region.^26^ The role of pharmacists as immunizers is still controversial and very limited data are available.^27^ The FIP reported in 2016 that Lebanese pharmacists do not tend to promote immunization, and initiatives around vaccination generally focus on the seasonal flu vaccine. In addition, patient awareness and promotion campaigns are very limited with a lack of national pharmacy‐immunization awareness campaigns.^28^ This is possibly attributed to major challenges and barriers, and lack of legislation updates on the scope of Lebanese pharmacy practice to include immunization services. This study aimed to determine barriers, and assess challenges and possible opportunities to provide immunization in the Lebanese pharmacy practice.

## 2 METHODS

### 2.1 Study design and participants

This was a cross‐sectional study that included pharmacists from all over Lebanon, as part of a national research to assess the role of pharmacists as immunizers. Data were collected through a snowball sampling technique utilizing an electronic questionnaire. To collect a sample from different districts all over Lebanon, the questionnaire was also shared on the Order of Pharmacists of Lebanon smart phone application with all registered pharmacists. The questionnaire form included a cover section with detailed information to explain the objective and context of the study. All registered pharmacists in Lebanon practicing in community, hospital or other clinical settings were considered eligible for participation. The pharmacists needed around 20 min time to complete the questionnaire.

### 2.2 Questionnaire and outcomes

A web‐based self‐administered validated questionnaire that is initially developed by the American Pharmacists Association was adapted with permission.^29^ Reliability testing was performed. The internal consistency of the questionnaire showed a good Cronbach's alpha of 0.717 among our sample.^30^ A total of 50 questions were included over 5 parts of the questionnaire. The first part involved the sociodemographic characteristics of pharmacists. The second part assessed whether the pharmacist has completed an injection and immunization training program or not. Pharmacists who completed any immunization training program were asked about their level of confidence to provide vaccination based on the certificate they acquired. They were also asked about what should be added for these programs to better prepare pharmacists for providing immunization. Pharmacists who did not complete any immunization training program were asked about their willingness to complete any of these training programs in the future.

The third part included questions about services offered at the participant's pharmacy. This part identified barriers to provide immunization in pharmacy practice. It allowed the participant to select all options that apply around reimbursement concerns, lack of pharmacy space to store and/or to administer vaccines, lack of support from management and/or physicians, time needed for professional development or additional training, costs associated with professional development and/or additional training, liability and malpractice concerns, personal belief that it would not serve the public, insufficient man power, lack of confidence about recognizing adverse events following immunization, lack of knowledge to manage adverse events following immunization, record keeping, and personal thoughts that the benefits do not outweigh the risks.

In the fourth part, immunization services provided by the pharmacist including vaccine administration (type, frequency, and duration of providing this service), patient education, and management of adverse events were assessed. The fifth part included questions about recognition for immunization services. Five‐point Likert scales were used to assess pharmacist recognition by other healthcare providers, public health stakeholders and patients as an immunizer; as well as the pharmacists' beliefs about the needs that should be addressed to expand successfully the immunization services provided.

### 2.3 Sample size calculation

The minimum sample size was calculated using CDC's Epi Info version 7.2.4. for population surveys. The expected frequency that yields the larges sample size was used to allow for adequate power of statistical analysis, and to produce a 95% confidence level with an acceptable margin of error of 5%. A minimum of 240 participants were required to assess barriers in providing immunization services based on expected frequency of 80%.^29^ This minimal size allowed to assess for the additional outcomes as they had expected frequencies between 90% and 93%, and therefore required a smaller sample size to be evaluated.

### 2.4 Ethical aspect

The Ethics and Research Committee of the School of Pharmacy at the Lebanese International University approved the protocol (2020RC‐033‐LIUSOP approved December 9, 2020). Informed consent was obtained by all participants, as they should have agreed to participate before being able to fill the survey.

### 2.5 Statistical analysis

Data were analyzed using IBM Statistical Package for Social Sciences (IBM SPSS) version 26.0. Frequencies and percentages were used to report the categorical variables. Chi‐square was used to compare all variables in the bivariate analysis. Multivariable analysis using binomial logistic regression was performed for all variables with *p* value lower than 0.2 in the bivariate analysis. The multivariable analysis evaluated barriers encountered in pharmacy practice as the independent predicting variables on administering vaccines as the dependent variable. The results were reported as adjusted odds ratios (ORa) and 95% confidence interval (CI), with a level of significance set at *p* ≤ 0.05.

## 3 RESULTS

### 3.1 Pharmacists' experiences and perception

A total of 315 pharmacists responded to the survey. Most of the participants were females (79.4%), aged between 20 and 29 years (74.9%), hold PharmD degree as the highest level of education (49.5%), and had 1–4 years of experience (41.0%). Only 24.1% declared completing an immunization training program, yet 90.9.% plan to complete the program in the future. Most of the reasons for not completing an immunization training program were lack of support from employer (21.43%), do not feel it is part of a pharmacist's scope of practice (15.48%), no enough reimbursement (14.29%), and being afraid of needles (15.48%). The majority of those who completed the program (90.6%) are confident to very confident in providing immunization based on the program certification, 43.2% believe that the current program is sufficient to prepare pharmacists, and 45.9% believe that the program can be expanded by live day workshops to better prepare pharmacists to provide immunization. The majority of participants (75.2%) also appear to be up‐to‐date with their personal immunization, and 78.7% receive the annual influenza vaccine. Table 1 reports the detailed experiences and perception of pharmacists related to immunization, and immunization services.

**Table 1:** Experiences and perception of pharmacists related to immunization, and immunization services.

Variable	*N* (%)
Completion of immunization training program	
▪ No	239 (75.9)
▪ Yes	76 (24.1)
Plan to complete immunization training program in the future	
▪ No	23 (9.1)
▪ Yes	230 (90.9)
Level of confidence to provide immunization after completion of training program	
▪ Very confident	42 (56.8)
▪ Confident	25 (33.8)
▪ Neutral	6 (8.1)
▪ Not confident	1 (1.4)
What should be added to completed training program to better prepare pharmacists to provide immunization?	
▪ Expand live day workshop	34 (45.9)
▪ Expand online modules	8 (10.8)
▪ Nothing, current program is sufficient to prepare pharmacists	32 (43.2)
Are you up‐to‐date with personal immunization?	
▪ I don't know	42 (13.3)
▪ No	36 (11.4)
▪ Yes	237 (75.2)
Do you receive the annual influenzae vaccine?	
▪ No	67 (21.3)
▪ Yes	248 (78.7)
Do you administer vaccines to patients in your practice?	
▪ No	129 (41.0)
▪ Yes	186 (59.0)
How long have you been administering vaccines?	
▪ <12 months	67 (36.0)
▪ 13–24 months	38 (20.4)
▪ 25–36 months	29 (15.6)
▪ 37–48 months	10 (5.4)
▪ > 48 months	42 (22.6)
How does your pharmacy offer immunization services?	
▪ A combination of appointments and walk‐ins	27 (8.6)
▪ Appointment only	18 (5.7)
▪ Walk‐in anytime	82 (26.0)
▪ Walk‐in during certain times/seasons of the year (e.g., flu shot)	90 (28.6)
▪ Walk‐in during designated times and days	9 (2.9)
▪ We do not offer immunization services	89 (28.3)
Where are vaccines administered at your pharmacy?	
▪ Private room	162 (60.4)
▪ Public area	10 (3.2)
▪ Semi‐private area	96 (35.8)
Approximate number of vaccines you administered last year	
▪ <50	102 (54.8)
▪ 50–100	58 (31.2)
▪ 101–150	13 (7.0)
▪ 151–200	7 (3.8)
▪ >200	6 (3.2)
Have you managed serious adverse events (such as allergy) related to immunization?	
▪ No	165 (88.7)
▪ Yes	21 (11.3)
Do you educate the public about immunization?	
▪ Often—always	189 (60.0)
▪ Sometimes	75 (23.8)
▪ Rarely—never	51 (16.2)

### 3.2 Immunization services

Over half of pharmacists (*N* = 186, 59.0%) administer vaccines to patients, and 67 (36.0%) have been providing this service for less than 12 months. The vaccines administered by pharmacists are mainly influenza and tetanus vaccines. More than half of the pharmacists (*N* = 102, 54.8%) also reported administering less than 50 vaccines within the last year, and only 21 (11.3%) managed serious adverse events related to immunization including allergic and hypersensitivity reactions. The pharmacists indicated that their pharmacies offer immunization services through walk‐in anytime (*N* = 82, 26.0%) or walk‐in during certain seasons (e.g., flu shot) (*N* = 90, 28.6%), and 162 (60.4%) pharmacies administer vaccines in a private room. Greater than half of participants (*N* = 189, 60.0%) often to always educate the public about immunization (Table 1).

### 3.3 Barriers to providing immunization

The major barrier to provide immunization was lack of support from physicians to pharmacists, perceived by over half of the pharmacists (*N* = 180, 57.1%), followed by the time needed for professional development and training (*N* = 110, 34.9%). Additional important barriers included reimbursement concerns (*N* = 75, 23.8%), lack of pharmacy space to store vaccines (*N* = 80, 25.4%), lack of space to administer vaccines (*N* = 78, 24.8%), lack of support from management (*N* = 70, 22.2%), cost associated with professional development and additional training (*N* = 67, 21.3%), and liability and malpractice concerns (*N* = 64, 20.3%).

### 3.4 Bivariate analysis of barriers on administering vaccination in pharmacy practice

There was a significant positive association between lack of support from physicians and the frequency of vaccine administration (66.7%) by pharmacists (*p* = 0.001). There was also a significant negative association between the barrier of cost associated with professional development and additional training, and the frequency of vaccine administration (44.8%) by pharmacists (*p* = 0.007). No significant other significant positive or negative associations were found between other identified barriers and the frequency of vaccine administration. The complete bivariate analysis of barriers to administer vaccination is reported in Table 2.

**Table 2:** Bivariate analysis of barriers to administer vaccination in pharmacy practice.

	Do not administer vaccines to patients	Administer vaccines to patients	
	*N* = 129 (41.0%)	*N* = 186 (59.0%)	
Characteristic/variable	*N* (%)	*N* (%)	*p* value
Reimbursement concerns			0.939
▪ No	98 (40.8)	142 (59.2)	
▪ Yes	31 (41.3)	44 (58.7)	
Lack of pharmacy space to store vaccines			0.950
▪ No	96 (40.9)	139 (59.1)	
▪ Yes	33 (41.3)	47 (58.8)	
Lack of pharmacy space to administer vaccines			0.061
▪ No	90 (38.0)	147 (62.0)	
▪ Yes	39 (50.0)	39 (50.0)	
Lack of support from management			0.232
▪ No	96 (39.2)	149 (60.8)	
▪ Yes	33 (47.1)	37 (52.9)	
Lack of support from physicians			0.001
▪ No	69 (51.1)	66 (48.9)	
▪ Yes	60 (33.3)	120 (66.7)	
Time needed for professional development/additional training			0.153
▪ No	78 (38.0)	127 (62.0)	
▪ Yes	51 (46.4)	59 (53.6)	
Costs associated with professional development/additional training			0.007
▪ No	92 (37.1)	156 (62.9)	
▪ Yes	37 (55.2)	30 (44.8)	
Liability/malpractice concerns			0.427
▪ No	100 (39.8)	151 (60.2)	
▪ Yes	29 (45.3)	35 (54.7)	
Do not feel it would serve the public			0.481
▪ No	122 (40.5)	179 (59.5)	
▪ Yes	7 (50.0)	7 (50.0)	
Insufficient man power			0.124
▪ No	120 (40.0)	180 (60.0)	
▪ Yes	9 (60.0)	6 (40)	
Lack of confidence about recognizing adverse events following immunization			0.106
▪ No	107 (39.2)	166 (60.8)	
▪ Yes	22 (52.4)	20 (47.6)	
Lack of knowledge of management of adverse events following immunization			0.706
▪ No	109 (40.5)	160 (59.5)	
▪ Yes	20 (43.5)	26 (56.5)	
Record keeping			0.172
▪ No	111 (42.7)	149 (57.3)	
▪ Yes	18 (32.7)	37 (67.3)	
I do not think the benefits outweigh the risks			0.119
▪ No	122 (40.1)	182 (59.9)	
▪ Yes	7 (63.6)	4 (36.4)	

### 3.5 Multivariable analysis of barriers on administering vaccination in pharmacy practice

There was a significant association between lack of support from physicians and cost associated with professional development and additional training, and vaccine administration. Pharmacists who identify lack of support from physicians as a barrier had higher odds of vaccine administration (ORa = 2.099, 95% CI = 1.290–3.414, *p* = 0.003). On the other hand, pharmacists who identified cost of professional development as a barrier had lower odds of vaccine administration (ORa = 0.533, 95% CI = 0.287–0.989, *p* = 0.046). There was no significant association with the other barriers that were included in the model (Table 3).

**Table 3:** Multivariable analysis of barriers to vaccine administration in pharmacy practice.

Variable	Adjusted OR	*p* value	95% CI	
			Lower	Upper
Lack of pharmacy space to administer vaccines	0.779	0.378	0.447	1.357
Lack of support from management to pharmacists	0.706	0.238	0.395	1.259
Lack of support from physicians to pharmacists	**2.099**	**0.003**	**1.290**	**3.414**
Time needed for professional development/additional training	0.839	0.517	0.492	1.428
Costs associated with professional development/additional training	**0.533**	**0.046**	**0.287**	**0.989**
Insufficient man power	0.622	0.415	0.198	1.949
Lack of confidence about recognizing adverse events following immunization	0.653	0.244	0.319	1.337
Record keeping	1.773	0.102	0.893	3.520
I do not think the benefits outweigh the risks	0.435	0.202	0.121	1.561

*Note*: Values in bold are statistically significant.

Abbreviations: CI, confidence interval; OR, odds ratio.

### 3.6 Recognition of pharmacists as immunizers and expansion of their role

Less than one‐third of pharmacists (28.5% agree to strongly agree) felt that they are fully accepted by the local public health authorities as immunization providers. Around one‐quarter (26.1%) felt that they are fully accepted as immunizers by local physicians, 54.5% by patients, and 51.8% by the pharmacy management. Pharmacists reported logistic, financial, and legislative requirements as essential necessities to successfully expand pharmacist‐led immunization services. The pharmacists considered that the provision of immunization services necessitates access to vaccines (56.6%), financial compensation for the service (41.9%), and recognition by payers (36.5%); as well as the support from local physicians (38%), the pharmacy management (42.2%), and public health stakeholders (46.1%). Further necessities reported by the pharmacists included additional immunization training (57.5%), integration of vaccination within the existing patient care services (32.6%), and access to patient medical record (50.6%).

## 4 DISCUSSION

The current study investigated the apparent barriers, challenges and possible opportunities of Lebanese pharmacists to offer immunization services, in the presence of legislative gaps in describing the role of pharmacists as immunizers. It included pharmacists practicing in community and noncommunity settings, and assessed their completion to an immunization training program and the challenges that they encounter in providing immunization services within their practice settings. More than half of the respondents were administering vaccination, commonly influenza and tetanus vaccines. However, the majority perceived themselves not recognized as immunizers by other providers and public health stakeholders.

We found that seasonal influenza vaccination is the main immunization service provided to the community by pharmacists. Most pharmacies provide walk‐in immunization services during the flu shot season. Although pharmacists are not reimbursed for this service as the patient usually self‐pay for the vaccine product with no additional cost for the service. Isenor and colleagues also found that seasonal influenza vaccine is the most frequently administered vaccination in New Brunswick, Canada.^29^ However, the Canadian pharmacists are reimbursed for influenza immunization services within a publicly financed immunization program.^31^ In addition, pharmacists frequently administer several vaccines including pneumococcal, herpes zoster, and Hepatitis A and B, all of which are patient self‐paid or covered by private insurance.^29^, ^32^

This study found that the most frequently perceived barriers in the provision of immunization are related to limited support, logistics and resources. Surprisingly, we found a significant positive association between lack of support from physicians and vaccine administration. Pharmacists who perceive lack of physicians' support as a substantial barrier were administering more vaccines compared to other pharmacists. This finding could be the result of ongoing debate and conflicts between local physicians and pharmacists on the scope of practice and the authority to provide immunization.^33^ Indeed, there is an argument between Lebanese physicians and pharmacists on the role of each healthcare profession in the provision of immunization services. While pharmacists are aiming to get well‐positioned in the healthcare system and seek to establish their role as immunizers, they are expecting a greater support from physicians on this scope of practice. Lebanese pharmacists perceive lack of support from physicians as a barrier to provide immunization, although the current findings showed higher rates of pharmacist‐led vaccination in the context of lack of support of physicians. Therefore, it is suggested that this remains a barrier as more pharmacists would engage in immunization services in light of better support from physicians and other healthcare members and stakeholders.

We also found a negative association between costs that are associated with professional development and additional training, and vaccine administration. Our results are consistent with other findings that reported financial resources as a principal barrier in providing vaccination services. In addition to restricted access to publicly financed vaccines, lack of well‐trained pharmacy staff, and space limitations.^22^, ^23^, ^24^, ^32^ In fact, challenges relating to the vaccination systems and processes have been globally addressed. Recommendations to overcome such barriers focus on enhancing the vaccination infrastructure,^34^ upgrading legislation and vaccination policies,^35^ obtaining political commitment to enhance vaccine regulation,^36^ implementing fair reimbursement systems,^37^ and enhancing and strengthening vaccination surveillance systems.^35^, ^38^, ^39^

Over half of the pharmacists perceive themselves completely recognized as immunizer by patients. This is justified by the high frequency of educational activities that Lebanese pharmacists offer to the public around immunization. Similar findings were reported by a public survey on the potential pharmacists' role as immunizers.^40^ The qualification of being a pharmacist could provide initial aptitude for vaccination provision, although a previous study from Lebanon reported that continuous education and training workshops are a main element that is needed for implementing immunization services by Lebanese pharmacists.^41^ This could be a great opportunity for pharmacists to expand their role as immunizers and vaccine services providers. In spite of this positive public attitude, targeted efforts on Lebanese physicians and public health authorities are demanded for complete acceptance of pharmacists in the provision of vaccination and expansion of the national immunization level, as other research has reported much higher acceptance rate by physicians and health departments.^29^ Indeed, community pharmacies are a first point of contact for patients because of their accessibility and distribution, providing a great opportunity to extend and enhance access to medication and vaccination.^42^ Another important opportunity to the pharmacists and benefit to the public are the convenient operating hours of community pharmacies, which may be highly appealing to both working and nonworking people.^43^ This is especially essential in rural, remote, and medically underserved regions, where access to vaccination centers might be difficult.^28^, ^44^ In the United States, vaccination rates doubled among young adults after 10 years of implementing pharmacy‐based vaccination, highlighting the benefits of the service provided.^45^ Moreover, the role of pharmacists as educators, facilitators and immunizers is especially imperative amid pandemics (e.g., influenza, COVID‐19) and other challenging circumstances that could significantly influence the morbidity and mortality of the general population.^46^, ^47^, ^48^, ^49^ Indeed, pharmacists reportedly can support immunization programs in challenging times and within pandemics if they are provided with the necessary recognition and autonomy.^50^

Nevertheless, there is no current standardized immunization training programs for pharmacists in Lebanon. In fact, the current completion of immunization training by Lebanese pharmacists could be an elective or additional training for continuing education and professional development. The lack of clear legislation on the role of pharmacists as immunizers may have been associated with fewer pharmacists completing these training programs. Although our findings showed that the majority of pharmacists have the plan to complete an initial or additional immunization training, a considerable number of pharmacists were found to provide immunization services without any training. Consequently, the current findings warrant urgent interventions from stakeholders to upgrade the healthcare strategies and legislative policies to specify the role of pharmacists as immunizers. Furthermore, the upgraded healthcare strategies and policies should identify compulsory trainings for pharmacists to have the necessary skills and experiences to provide immunization services, and thus minimize the drawback of malpractices from nontrained pharmacists.

This study has several strengths. It is the first study to investigate challenges and opportunities of Lebanese pharmacists to expand their role as immunizers. The research instrument that was used had a good internal consistency testing, and thus providing good reliability of our findings. The results provide insight for discussion with public health stakeholders and policy makers to empower pharmacists and expand their scope of practice to provide immunization services. On the other hand, the main limitation of the study is the cross‐sectional design that cannot assess temporality, and therefore, could not reflect how challenges and opportunities are changing with the drastic socioeconomic crisis that is limiting medical and societal resources in Lebanon for more than 1 year.^51^ The continuous deterioration of financial conditions and value of local currency may be residual confounders to the cost of professional development and additional training of pharmacists. Another limitation is a possible response bias due to the electronic self‐administration of the study questionnaire. This method of data collection may have excluded certain pharmacists that would consider additional barriers that are related to digital resources. Moreover, the snowball sampling technique is associated with a possible risk of selection bias. It may have directed the sample toward a consistent subgroup of pharmacists that are up‐to‐date with their personal immunization and therefore good immunization advocates despite lack of adequate immunization training programs. Another risk of selection bias may be related to the region of practice. However, it is believed that this bias is minimized as the sample was collected from different districts all over Lebanon. Last, the study included young pharmacists mainly, therefore the findings cannot be generalized for senior pharmacists.

## 5 CONCLUSIONS

Pharmacists in Lebanon are active contributors to immunization services. They are well positioned in the community to perceive themselves supported by patients for this role. Nevertheless, the scope of pharmacy practice is not recognized by other healthcare providers and stakeholders to include immunization services. The major barriers to vaccine administration by pharmacists comprised lack of physicians' support and expenses associated with professional development and additional training. Pharmacists administer more vaccination with the lack of support from physicians; whereas they administer less vaccination due to cost associated with professional development and further training. Additional research is suggested in this scope to determine the mediator role of practice challenges on the provision of immunization, and to provide barrier‐tailored recommendations to overcome the encountered difficulties.

## AUTHOR CONTRIBUTIONS

**Fouad Sakr**: conceptualization; formal analysis; investigation; methodology; resources; software; writing—original draft. **Mariam Dabbous**: writing—review & editing. **Mohamad Rahal**: writing—review & editing. **Pascale Salameh**: supervision. **Marwan Akel**: conceptualization; resources; supervision.

## CONFLICT OF INTEREST STATEMENT

The authors declare no conflicts of interest.

## TRANSPARENCY STATEMENT

The lead author Fouad Sakr affirms that this manuscript is an honest, accurate, and transparent account of the study being reported; that no important aspects of the study have been omitted; and that any discrepancies from the study as planned (and, if relevant, registered) have been explained.

## Data Availability

The datasets analyzed in the paper are available from the author or corresponding author upon readers' requests.
